# Time for Space and the Stability of Prospective Control: Reaching-to-Grasp Gibson

**DOI:** 10.1177/20416695211054533

**Published:** 2021-11-06

**Authors:** Geoffrey P. Bingham

**Affiliations:** Department of Psychological and Brain Sciences, Indiana University, Bloomington, IN, USA

**Keywords:** prospective information, visually guided reaching, proportional rate control, stability, neural transmission delay

## Abstract

Gibson formulated an approach to goal-directed behavior using prospective information in the context of visually guided locomotion and manual behavior. The former was Gibson's paradigm case, but it is the rapidity of targeted reaching that has provided the special challenge for stable control. Recent treatments of visually guided reaching assume that internal forward models are required to generate stable behavior given delays caused by neural transmission times. Internal models are representations of the sort eschewed by Gibson in favor of prospective information. Reaching is usually described as guided using relative distances of hand and target, but prospective information is usually temporal rather than spatial. We describe proportional rate control models that incorporate time dimensioned prospective information and show they remain stable in the face of delays. The use of time-dimensioned prospective information removes the need for internal models for stable behavior despite neural transmission delays and allows Gibson's approach to prevail.

In *The ecological approach to visual perception* (*EAVP*), Gibson (1979) discussed two particular tasks at length, namely, visually guided locomotion and visually guided manual action. However, visually guided locomotion was his paradigm case. His treatment of it was much more developed than that of visually guided reaching, which is not so surprising when you consider that his analysis of visual information was almost exclusively monocular. He provided a reasonably detailed description of the monocular optic flow patterns in the optic array for the locomoting observer (*EAVP*; Gibson, 1979, pp. 121–126). When it came to visually guided reaching, he described the optical minification of the squirming five-pronged shape or silhouette that specifies the hand and its extension on the end of the arm in reaching out (*EAVP*; Gibson, 1979, pp. 120–121). A similar description of this monocular information that could be used to guide a targeted reach appears in Herth et al. (2021), where the information was assessed as not so effective. Alternatives were then offered that will be described and discussed below.

## Goal-Directed Behavior: Representations Versus Prospective Information:

Gibson's thinking about perception was most often with an eye to goal-directed behavior, which as future-directed, provides a special challenge for psychology's scientific aspirations. Intended future states cannot directly determine past actions performed to achieve those states. A popular solution has been to assume that descriptions of those goals are represented internally and that those representations are given causal responsibility for the actions performed to attain the goals. Gibson did not favor this solution. Instead, he offered the idea of information that is future-directed or prospective and that could be detected in perception used to control goal-directed action. (See Reed (1988) and Reed and Jones (1982) for the development of Gibson's thinking and Warren et al. (2005) for extended discussion in the context of direct perception as well as Zhao and Warren (2015) on internal models.) The ideas were nascent in 1979 and not extensively worked out and elaborated. A prime example was Gibson's steering strategy for approach in locomotion, which was to move to locate the Focus Of Expansion (FOE) in the optic radial outflow within the visual solid angle (or image or optical patch) projected from the surface to be approached (*EAVP*; Gibson, 1979, pp. 225–234). Gibson's radical approach was to skip the world model and act to control the optics as such. Move to produce the relevant spatio-temporal optical pattern and then to maintain it to achieve the intended goal. The particular optical pattern is prospective if maintained.

## Monocular Tau and Constant Tau-Dot Control

Another example where Gibson addressed the problem of anticipation or prediction of future states was the information for imminent collision or looming (*EAVP*; Gibson, 1979, p. 231). This was image expansion at an exponentially increasing rate. Although it had been developed by Hoyle (1957) and Lee (1974, 1976, 1980), the optical variable Tau did not appear in *EAVP* as specifying Time-to-Collision (or Time-to-Contact) under conditions of a constant velocity of approach. Analytically, Tau is the ratio of image size to image expansion rate or (using the FOE as a natural coordinate origin) optical position over optical velocity. Tau is the best-known example of prospective information (first referred to specifically as such by Lee), predicting as it does when an approaching surface will hit the observer. Extreme sensitivity to Tau as such was demonstrated in human vision by Todd (1981) and Regan and Hamstra (1993). The use of Tau-related information in a classic Gibson strategy for control of deceleration in approach behaviors was formulated by Lee (1976). The strategy was to use the rate of change of Tau, Tau-dot, and to move to produce and then maintain Tau-dot  =  −0.5 in the optical pattern projected from a surface being approached. The advantage of Tau-dot  =  -0.5 is that it corresponds to constant deceleration to stop right at the target. Thus, once Tau-dot  =  −0.5 has been achieved, the agent only needs to maintain it, as suggested by Gibson, to achieve the goal safely.

However, two major problems with the constant Tau-dot strategy were subsequently described by Fajen (2005a, 2005b, 2005c, 2007). The first was that the strategy required the production of a single, unique value, namely, −0.5. This lack of flexibility implied a lack of stability. What if that value could not be produced? Fajen pointed out that the strategy did not include information as to when braking should be initiated given the braking capability under the circumstances, that is, the affordance for braking of the perceiver. This was the second problem.

## Information-Based versus Affordance-Based Control

As part of his approach to goal-directed behavior in terms of prospective information, Gibson described the perceptible properties of the surroundings as affordances (*EAVP*; Gibson, 1979, pp. 127–128). Affordances are action-relevant properties of surfaces, objects, and events related to the action capabilities, or effectivities (Turvey et al., 1981), of the perceiver. As perceptible properties, affordances entail information that is about possible future states, possible future actions (what Gibson referred to as potential actions), and goals achieved through them (*EAVP*; Gibson, 1979, pp. 140–141).

Fajen contrasted information-based and affordance-based control and formulated an instance of the latter to address the problems with the Tau-dot strategy. He argued that braking should be controlled using information about current deceleration relative to ideal deceleration and used to keep braking within the bounds of the capability. This formulation does not predict specific trajectories and thus differs from Gibson's strategy, that is, information-based control, which entails a dynamic that generates specific trajectories, for example, constant Tau-dot. All of the information variables in this discussion were monocular and for visually guided locomotion, that is, controlling the trajectory of the eye (in a head on a body) through the environment.

## Visually Guided Reaching and Proportional Rate Control

Many studies had also applied monocular Tau to control manual action in interception tasks (e.g., Bingham, 1995; Bootsma and Oudejans, 1992), that is, reaching-to-grasp, catching, or hitting balls in table tennis. Anderson and Bingham (2010, 2011) pointed out that monocular Tau is only relevant to approach and contact with the eye, not the hand (see also Wann et al., 1993). With this, they formulated an approach to the visual control of reaching-to-grasp that was in contrast to both the constant Tau-dot strategy and Fajen's affordance-based control, although this new formulation satisfied the need to respect the relevant affordances while generating stable trajectories in conformance with Gibson's strategy. This approach entailed the introduction of new Tau-type visual information variables together with a new type of control dynamic, proportional rate control.

## New Tau Variables

Inspired by Gibson's descriptions and discussions about (monocular) optic flow, an extended debate developed in the 1970s around numerous studies investigating visual motion measurement to address the question: is motion inferred and computed from measured displacements or is motion measured directly, for example, as motion energy? The conclusion was that the motion that comprises optic flow is measured directly. A reprise of this debate ensued in the 1990s but this time addressing possible stereo motion and its measurement via two different channels, namely, CDOT or Change of Disparity Over Time (disparity first, then the derivative) and IOVD or Inter-Ocular Velocity Difference (derivative first, then disparity). The latter entails inter-ocular disparities between (monocular) optic flow vectors. The accumulated evidence has indicated that there are three interrelated motion measurement channels in the visual system (see Patterson, 1999; Rokers et al., 2008, 2009 for a review). The existence of the latter two stereo channels encouraged the formulation of disparity Tau variables in alternate forms. One described by Grey and Regan (1998, 2004) is comparable to (although slower than) monocular Tau in that it is about time to contact with the eyes (Fath et al., 2018). In contrast, Anderson and Bingham (2010) formulated another disparity Tau hypothesized to be used to perceive time-to-contact with the hand in the context of reaches to grasp. This Tau is time to achieve disparity matching which had been shown to be used to guide target acquisition in reaches to grasp (Anderson & Bingham, 2010; Bingham et al., 2001; Melmoth & Grant, 2006).

Additional monocular Tau variables have been formulated in the context of visually guided reaching, ones involving the viewing geometry in reaching to grasp an object across a supporting surface like a table or counter (Herth et al., 2021). Unlike the original monocular Tau, these are about the contact of hand and target object. For instance, Herth et al. described a monocular Tau variable composed of differential optical texture projected from the support surface visible beneath the edges of the hand and the target object, respectively. This is one of a number of such variables formulated by these authors (Anderson & Bingham, 2010; Bingham, Herth, Pin, Chen, & Wang, submitted a; Bingham, Wang, & Herth, submitted b).

## Proportional Rate Control

Proportional rate control entails a control dynamic specific to each of the different Tau variables (Fath et al., 2014; Herth et al., 2021). General to them all, however, is control to establish and maintain a selected value for the ratio of the particular Tau-dot to Tau variable. That is, the rate of change of Tau as it decreases with an approach to a target is kept in proportion to Tau itself, so that when Tau is large, far from the target, the rate of change is larger and as Tau gets small, the rate of change also becomes small. The resulting dynamic is extremely stable. It has been found to spontaneously recover, for instance, when the available braking fails to meet the momentary deceleration requirement (Fath et al., 2013). Beyond this, the flexibility provided by the fact that different proportional rate constants yield different approach times (as would be required to model fast and slow reaching) means that if an intended constant proportional rate cannot be achieved, then a less demanding one can be used to yield successful approach to stop at the target. Next, the dynamic yields behavioral trajectories that are much more general than mere deceleration. That is, the dynamic generates entire representative reach trajectories including both the accelerative and decelerative phases. Finally, the associated strategy addresses the affordance problem in the case of braking. Before braking has begun, Tau-dot is −1 so the ratio is determined by the value of Tau. A given value of Tau can be calibrated with respect to the braking capability and used to initiate braking when the ratio reaches that value which is also then maintained as the intended (calibrated) constant proportional rate (see Kadihasanoglu et al., 2010, 2015; Kadihasanoglu et al., 2021 for an extended discussion).

## Representations as the Basis of Stable Movements

Gibson expressed suspicion of the notion of feedback and argued instead that information is required for control of actions (*EAVP*; Gibson, 1979, p. 226). He observed that not all motions in actions are actively generated, some are passively imposed, but either way the actor must perceive what is happening to control goal achievement effectively. Feedback, he suggested, is only about actively or intentionally generated movements. Nevertheless, the problem of instability of feedback control in the context of delays imposed by neural transmission times has provided an argument for internal representations as essential to visually guided actions. The instability of feedback control is a well-recognized problem solved in classic engineering analyses by reducing the gain or strength of corrections to detected errors in proportion to the size of the delay (Franklin et al., 1994; Jagacinski & Flach, 2003). With the observation that visually guided reaches are typically performed in a very short time (≤1 s) and often exhibit a high-speed phase during which most of the distance to a target is traveled followed by a phase of slow speed adjustment, it was suggested that the initial high-speed phase is performed ballistically under strictly feedforward control while visual online guidance is used during the slow phase. However, “double-step” targeted reaching studies then showed that rapid adjustments were made smoothly and stably during the high-speed phase in response to change in target distance or direction (see Bingham, 1995 for a review). How was this possible given the expected instability with delay?

Miall et al. (1993) introduced the Smith-Predictor as a solution to this instability problem in which an internal feedforward model would generate predicted feedback with no delay. When this predicted feedback was combined with actual feedback in simulations, instability was reduced or eliminated. The existence and use of such internal feedforward models became widely accepted. Saunders and Knill (2003, 2004, 2005) performed a series of studies investigating extremely rapid (≈0.5 s) visually guided reaches in which the distance or direction of movement of the hand was perturbed during its high-speed movement. They used a virtual visual avatar for the hand to effect the perturbations. They reported rapid (≈100 ms) and stable adjustments that yielded accurate target acquisition by the hand. However, they concluded that internal models must have been the basis for this performance.

Both Zhao and Warren (2015) and Fajen (2021) have reviewed and critiqued the idea that internal models are used in visually guided locomotion, arguing instead for control using prospective information. However, in their critiques, they did not address the instability of control in the context of delays from neural transmission times used as an argument for internal models. On the other hand, visually guided manual actions, and in particular, reaches-to-grasp has been the application domain, rather than visually guided locomotion, in which this argument was developed because of the brief movement times exhibited in reaching.

## Trading Time for Space and Prospective Control

It has typically been assumed that relative distance information is used to guide targeted reaches, for instance, relative stereo disparity or declination angle, each of which entails an evolving difference in optical angles between the hand and target object. An example of a control dynamic that demonstrates the classic instability with representative neural delay times is one investigated by Bingham and Zaal (2004) in which relative distance information (i.e., relative disparity) was used to guide the virtual equilibrium point (EP) trajectory (Feldman et al., 1990) in an EP proportional–integral model for the control of reaching:

(1) $$\ddot{x}(t)+b\dot{x}(t)+kx(t)=k\int g\left(\frac{x_{T}-x(t)}{x_{T}}\right)dt$$

where *x*(*t*) is the distance or position of the hand and *x_T_* is the target distance or position.

**Figure 1.**
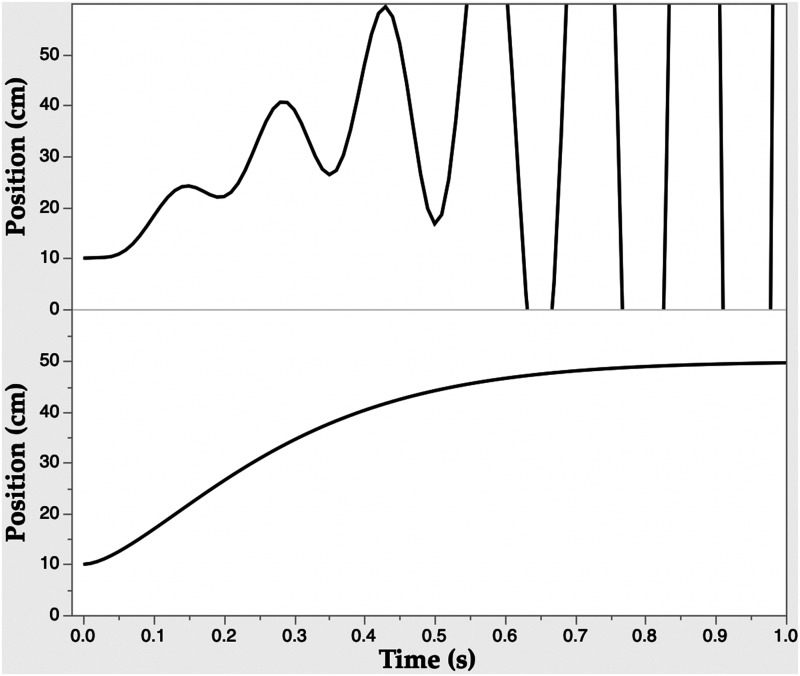
Simulations of reaches performed with an EP model driven by proportional–integral control using relative distance information. Lower panel: without information delay. Upper panel: with information delay of 30 ms.

The time series of hand position in a rapid (≈1 s) reach to a target at an initial distance of 40 cm is shown in Figure 1(bottom panel) without any delay of the relative distance information. It is representative of actual reaches. The time series for the simulated reach is shown in Figure 1(upper panel) with the relative distance information delayed by 30 ms in the model. The classic exponentially increasing instability occurs. This is just the behavior that an internal model would be invoked to repair. However, there is another possibility.

Because of the delay, the future must be predicted for stable behavior. Internal models can predict the future, but so does prospective information! A prospective control strategy has been to substitute time for space. For instance, Warren et al. (1986) modeled the control of running over irregular terrain by substituting perception of time-to-contact (by detecting differential Taus) for the perception of the distance between successive surfaces of the support. In the context of reaching, we substituted detection of disparity Tau^1^ for the perception of relative distance in a proportional rate dynamic used to generate virtual EP trajectories. The notable finding is that this time-based information variable remained stable in control of rapid movements despite delays simulating the effect of neural transmission times.

So, the EP model (equation (2)) was the same as in equation (1) except now the virtual EP trajectory, *y*(*t*), was determined by the proportional rate dynamic (equation (3)) for disparity tau (equation (4)):

(2) $$\ddot{x}(t)+b\dot{x}(t)+kx(t)=ky(t)$$

(3) $$\ddot{y}(t)=\left(\frac{\left(1-[2y(t)/x_{T}]\right)}{\tau_{\mathrm{Disp}}(t)}-P\right)\dot{y}(t)$$

(4) $$\tau_{\mathrm{Disp}}=\frac{x(t)}{\dot{x}(t)}\left(1-\frac{x(t)}{x_{T}}\right)$$

where *x*(*t*) is the hand position, *x_T_* is the target position, *y*(*t*) is the EP position, $\tau_{\mathrm{Disp}}$ is disparity Tau (see Anderson and Bingham, 2010), and *P* is the proportional rate constant: $d\tau_{\mathrm{Disp}}/dt=\;P\tau_{\mathrm{Disp}}$ . (See Herth et al. (2021) for the mathematical derivation of proportional rate equations similar to equations (3) and (4).) The proportional rate dynamic (equations (3) and (4)) looks a bit complex, but it simply brings the hand to the target by driving $\tau_{\mathrm{Disp}}$ to 0 by maintaining the ratio of $\tau_{\mathrm{Disp}}$ -dot to $\tau_{\mathrm{Disp}}$ constant and equal to *P*. The virtual EP trajectory, *y*(*t*), is generated by the dynamic of equation (3) using the visual information. Equation (4) describes the information detected online while *x_T_* is constant and detected before the reach is initiated. The value of P determines the speed of the reach.

**Figure 2.**
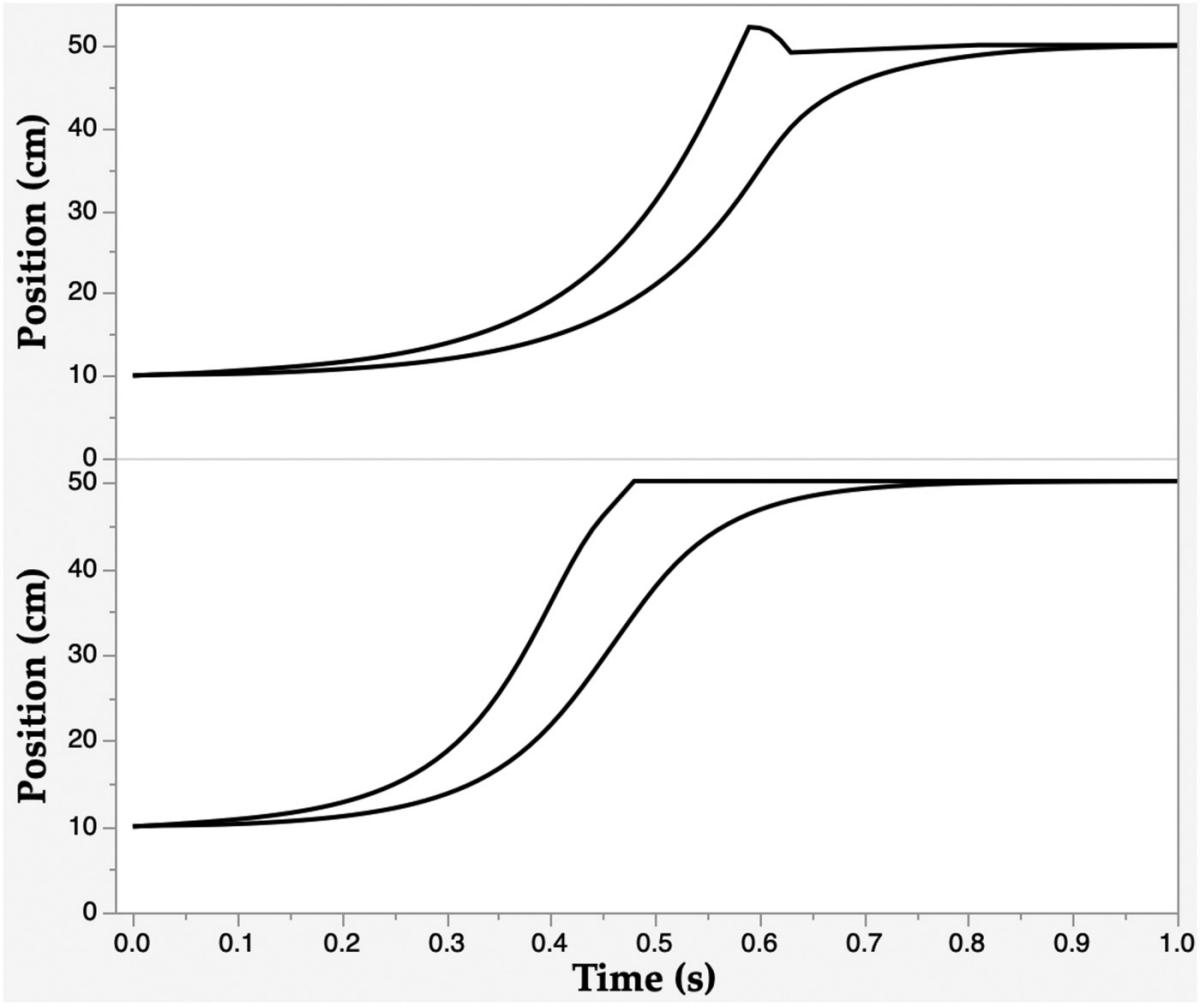
Simulations of reaches performed with an EP model driven by proportional rate control using disparity Tau information. Lower panel: without information delay. Upper panel: with information delay of 50 ms. In each case, the virtual EP trajectory reaches the target about 200 ms before the hand does.

As shown in Figure 2 in the lower panel, when the proportional rate model was used, without a delay, to simulate the same reach distance and timing as before, the trajectories were once again similar to actual reaches. Both the virtual EP and the hand trajectories appear in each plot. The virtual EP arrives at the target first followed by the hand. The upper panel shows the performance with a 50 ms delay of the visual information, that is, disparity Tau. The movement remains stable and is only shifted in the time to acquire the target by 100 ms. This remains stable with delays of 100 ms or greater. Control of reaches performed using time dimensioned prospective information is relatively unaffected by the hypothesized internal delays produced by finite neural transmission times.

Proportional rate control is an effective example of how prospective information can be used to visually guide and control actions without resort to internal representations, an example applicable to both fundamental types of actions discussed by Gibson, that is, reaching and locomotion. It addresses the problem of braking in a visually guided approach during locomotion. It also generates the entire trajectory for a reach including acceleration and deceleration phases. As Gibson suggested, by using prospective information instead of representations (or internal models), goal-directed behavior is able to charge into the future, stability and with confident intent, without looking back.

## Notes

1. Or the optical texture based Tau described by Herth, et al., (2021).
